# Amino Acid Profile of Fruits as Potential Fingerprints of Varietal Origin

**DOI:** 10.3390/molecules24244500

**Published:** 2019-12-09

**Authors:** Oana Romina Botoran, Roxana Elena Ionete, Marius Gheorghe Miricioiu, Diana Costinel, Gabriel Lucian Radu, Raluca Popescu

**Affiliations:** 1 National Research and Development Institute for Cryogenics and Isotopic Technologies–ICSI Rm. Valcea, ICSI Analytics Group, 4 Uzinei Street, RO-240050 Râmnicu Vâlcea, Romania; oana.dinca@icsi.ro (O.R.B.); marius.miricioiu@icsi.ro (M.G.M.); diana.costinel@icsi.ro (D.C.); raluca.popescu@icsi.ro (R.P.); 2 Faculty of Applied Chemistry and Materials Science, Politehnica University of Bucharest, 1-7 Polizu Street, 011061 Bucharest, Romania; gl_radu@chim.upb.ro

**Keywords:** amino acids composition, chemometrics, fruits, ^1^H-NMR

## Abstract

This study aims to assess the capability of the ^1^H-NMR profiling of fruits from different genera in combination with multivariate data analysis to provide feasible information for fruit juices’ authenticity in terms of botanical origin. Nine fruit varieties from four genera were selected for the experimental plan. The juice obtained from the fruits was characterized using the ^1^H-NMR technique, selecting the obtained amino acid profile of fruits as a potential specific fingerprint. Due to the complex information provided by the NMR spectra, a chemometric approach of the data was further applied to enable the differentiation of the fruit samples, highlighting thus its suitability as a discrimination tool for the varietal origin. The advantage of this analytical approach is given by the relatively simple working procedure, which consists of an easy, fast, and accessible preparation stage while providing complex information on fruit composition.

## 1. Introduction

Fruit juices, obtained by macerating or squeezing fruits, is one of the fastest growing segments in the beverages industry due to the increasing concerns among consumers for healthy products. A prevailing factor that influences the metabolic composition and quality of fruits, especially from a nutritional perspective due to the synergistic combination of antioxidants, phytochemicals, and dietary fibers, is the environmental growing conditions [1,2]. Each class of fruit juice has a unique chemical pattern characterized by primary metabolites (such as amino acids, sugars, organic acids), involved in basic cell functions, and secondary metabolites that are usually fruit-type specific. Considered together, these components set the main features of fruits, the nutritional value, aroma, taste, and beneficial effects on health. Therefore, they can be used as potential markers for quality, origin, and authenticity of fruit and fruit-derived foods [3].

Assessing and confirming the authenticity and quality of fruits and their by-products remains a challenging issue due to the plant/fruit metabolome complexity that limits the power of a single analytical tool to gather the entire metabolomics information. Thus, to get a whole image on both primary and secondary metabolites and other specific origin markers, several combined techniques should be merged. Alongside the conventional analytical approach using gas chromatography/mass spectrometry [4,5] and liquid chromatography/mass spectrometry [6,7], that also assume different extraction procedures, the nuclear magnetic resonance-based metabolomics [8,9] and stable isotopes investigation [10], along with chemometrics, are of interest for checking the conformity of fruit-based products.

The nuclear magnetic resonance (NMR) spectroscopy applied to fruit profiling may represent a reliable alternative in terms of a non-invasive and powerful method that combines targeted and untargeted analyses, since it can provide a picture, as complete as possible, of the sample chemical composition [9,11]. From a global perspective, the metabolomic analysis of ^1^H-NMR data from fruit extracts showed its ability in assessing the quality and authenticity of plant-based food [12], especially in terms of the botanical and geographical origin, comparison between different growing systems, chemical characterization, and identification of biological active compounds [3]. Also, the NMR-based metabolomic approach provides information regarding the relationship between the major metabolites and the sensory characteristics of the fruits. Nevertheless, the effects of genetic and environmental factors, and the interactive ecosystem in a given place, including soil, climate, and species, are essential in defining the metabolic profiles of fruits [13,14]. A study on apple juices originating from the same cultivar but different geographical origin [15] highlighted the different metabolic profile that distinguished the samples by provenance area, clarifying that the variables responsible for discrimination were mainly represented by several amino acids. It was shown that the metabolite content is a fingerprint of the fruit juice, reflecting the fruit’s environmental growing conditions, such as the seasonal and climatic factors and agricultural practices, providing thus reliable data to establish the geographic specificity (traceability) of the products [16]. Therewith, given the fact that the chemical composition of each individual plant is related to the genotype, the varietal origin of fruits could be defined using their unique metabolic profile.

With the globalization of the food trade and world food markets, the need of characterizing the specificity of local products, as well as the constantly increasing consumer demand for high quality foodstuff, has lead researchers to deepen the application of NMR metabolic profile in quality and authenticity characterization of food products, as priority areas. Due to its great potential applications in plant functional genomics, food science, and human nutrition, plant metabolomics becomes an evolving field and has been successfully applied to fruits [12,17,18]. Therefore, the aim of this work is to provide a better insight in the amino acids fingerprint of regional horticultural varieties using the ^1^H-NMR profiling as alternative technique. We intended to emphasize the relationship between the content of amino acids and the varietal origin of the fruit cultivar by a reliable, rapid, and non-difficult analytical approach, contributing thus to the portfolio of methods applied for the food traceability process. ^1^H-NMR spectra of juices are very rich in information, thus its combination with chemometric analysis revealed latent patterns in the data, which enabled the classification of the samples in terms of varietal provenance.

## 2. Results and Discussion

### 2.1. General Presentation of the ^1^H-NMR Spectra of Fruit Juices

**Figure 1:**
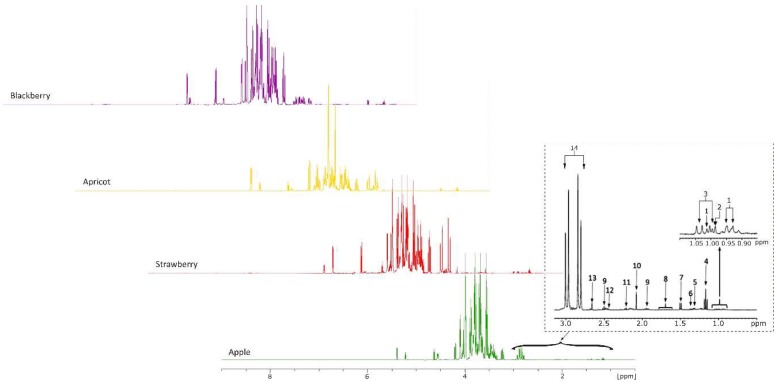
^1^H-NMR spectrum of four different fruit juices and typical examples of ^1^H expanded spectral region of amino acid region (0.5–3 ppm) of apple juice. **1**: Isoleucine; **2**: Leucine; **3**: Valine; **4**: Ethanol; **5**: Threonine; **6**: Lactic acid; **7**: Alanine; **8**: Arginine; **9**: Glutaric acid; **10**: Acetic acid, **11**: Glutamine; **12**: Pyruvic acid; **13**: Succinic acid; **14**: Citric acid.

In the ^1^H-NMR spectrum of fruit juices (Figure 1), a number of approximately 80 peaks can be observed and as expected, the spectra present wide regions with overlapping phenomena, which makes it difficult to proceed with the peak integration. The typical ^1^H-NMR spectrum of a juice shows three defined regions. In the first one, ranging from 0.5 to 3.0 ppm, the region of hydrogen atoms belonging to organic acids (citric and malic) and amino acids (alanine, valine, and proline) are present. The second region, between 3.0 and 6.0 ppm, is typical for carbohydrates, with sucrose, α-glucose, β-glucose, and fructose being the most abundant compounds, wherein hydrogens of anomeric carbons are clearly separated from the remaining sugar signals [16]. The last region, ranging from 6.0 to 8.5 ppm, reflect the phenolic metabolites and aromatic protons. Moreover, each juice presents the peak assigned to the methyl group of ethanol at 1.17 ppm [16,19]. Spectra of pure juice have similar shapes in the aliphatic region and limited quantitative differences, except for the two peaks at 5.22 and 4.47 ppm related to sucrose, which are present only in the blackberry juice. The region ranging from 6 to 10 ppm is meanwhile more typical; here, aromatic and phenolic compounds are normally present. In this case, the signals are lower in intensity with respect to the aliphatics. 

When the first region is analyzed, the intensity of citric acid signals (dd, 2.87 ppm) reveals that this compound far overcomes the other acids present in the juice sample. Moreover, it can be observed another signal of particular interest in this region of the spectrum, representing the triplet assigned to the CH_3_ group of the ethanol molecule (t, 1.17 ppm). The presence of this compound is generally associated with the unwanted alcoholic fermentation that naturally occurs when preparing the fruits for juice making. Even if all the spectra acquisition was performed immediately after the fruits juice preparation and pH adjustment, an amount of ethanol is always produced by the microorganisms present in the fruit [19].

Figure 1 highlights the fact that each individual fruit has a distinct set of primary metabolites, such as amino acids, sugars, and organic acids, which are involved in a series of basic functions of living cells. Generally, for the assignment of a specific compound in the complex spectra of fruit juice, at least one signal of this metabolite has to be resolved from other signals in the spectrum, due to the fact that not all metabolites give fingerprinting signals. There are certain situations when due to the very low concentration, the signal intensity of a particular compound is overlapped with the signal of another metabolite, mainly because the dispersion of the signals in the NMR spectrum is rather small, resulting in extensive overlap in the signals in most regions of the spectrum. At the same time, some metabolites, even if present in small quantities, have a characteristic signal (for example alanine, valine, isoleucine, etc.) and can be easily identified [3].

### 2.2. Amino Acid Profile: Assignment of the Interest Peaks and Statistical Elaboration

In particular, for this study, in order to investigate the correlation between amino acids metabolic information in fruits of various botanical origins (cherries, sour cherries, plums, apricots, peaches, apples, strawberries, raspberries, and blackberries), a number of 10 amino acids were identified, namely: Proline (Pro), arginine (Arg), lysine (Lys), asparagine (Asn), aspartate (Asp), glutamate (Glu), isoleucine (Iso), alanine (Ala), threonine (Thr), and valine (Val). In this type of NMR based analyses, the extraction procedure is probably the most critical step aimed to the quantitative transfer of the metabolites from the solid matrix into the solution. Since the sample preparation was based on a simplified procedure (maceration, centrifugation, filtration), there is the possibility to achieve a partial amino acid extraction, or for certain metabolites the extraction not to be performed at all. The obtained NMR peaks provide information regarding the proportion of soluble amino acids (free or from the peptide/protein structure) in the matrix and the ratio between them.

**Figure 2:**
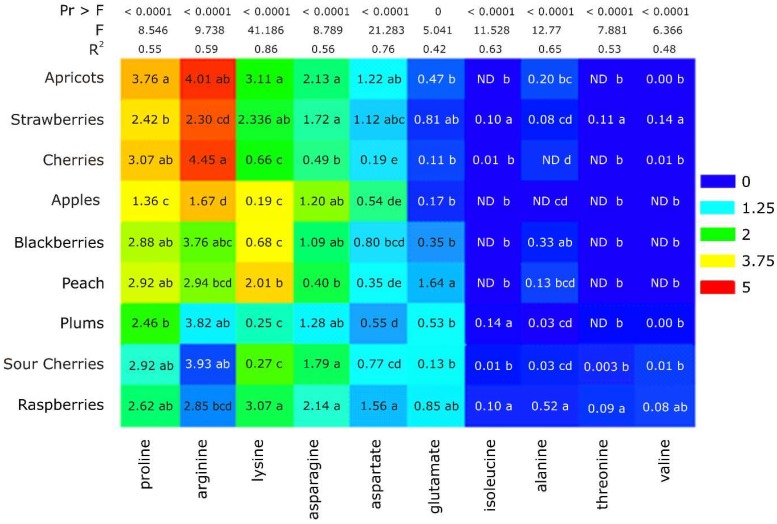
Heat map and amino acid composition of fruit juice samples according to their specific amino acid peaks in the ^1^H-NMR spectrum, as % of total NMR signals (values in the same row of Group 1 that are followed by different letters (**a**–**d**) differ significantly (*p* < 0.05)). Statistical analysis was one-way ANOVA with pairwise post hoc comparisons by the method of Tukey’s test.

A heat map was built (Figure 2), which also included the amino acid composition of fruit juice samples according to their specific peaks in the ^1^H-NMR spectrum, to better observe the existing differences in content between the ten important metabolites, for each fruit variety. It was remarked that almost all the identified amino acids had significant differences (*p* < 0.05) among the fruit juice varieties; only glutamate presented no significant difference. This could be due to the fact that the α-amino group allows assimilation and dissimilation of ammonia and is the building block of all other amino acids. In fruits, amino acids like arginine and proline are synthesized from glutamate and amongst the total free amino acids in ripened fruit, glutamate represents approximately 55% of the relative molar concentration, making it the main free amino acid, therefore it is not a fruit botanical origin specific marker. The heat map was also created to visualize the content composition in different fruits variety: Red, blue, and yellow areas indicate high, low, and moderate levels of metabolite composition, respectively. Apricots, strawberries, and cherries had the same relative contents: High Pro and Arg, moderate Lys, Asp, and Asn, and low content of the others.

A correlation analysis between the amino acid composition and botanical origin, based on the Pearson correlation coefficient was also used (Table 1). For this statistical approach, the fruit varieties were classified as individual species, but also according to their genus from which they belong, in order to observe additional correlations. The obtained values were between +1 and −1, where the numerical value indicates the correlation strength, and “+” or “−” indicates the positive or negative correlation. The variables correlated with *Prunus* and *Malus* genus were Pro and Arg, with all the correlation coefficients being > 0.45, whereas those positive correlated with *Fragaria* genus were Thr and Val, while for the *Rubus* genus a great correlation was with Ala [20]. Contents of particular amino acids are different for different juices, so they can be used as potential authenticity markers. For example, an increase content of Pro in raspberries or blackberries juice could indicate the additional presence of other fruit juices. Arg was the most abundant amino acid, on average, with the highest proportion being 4.45% of total NMR signals in the cherries juice samples, and the lowest proportion being 1.67% of total NMR signals in apple juice samples. While the proportions of amino acids observed in a juice sample from a given variety may be influenced by growing season [21,22], crop load [23], geographical origin, and other factors [16], our data provide insight as to the extent of variation for each amino acid, and the ranges of relative presence of amino acids that could be expected in different types of fruit juices.

### 2.3. Botanical Origin Discrimination

**Figure 3:**
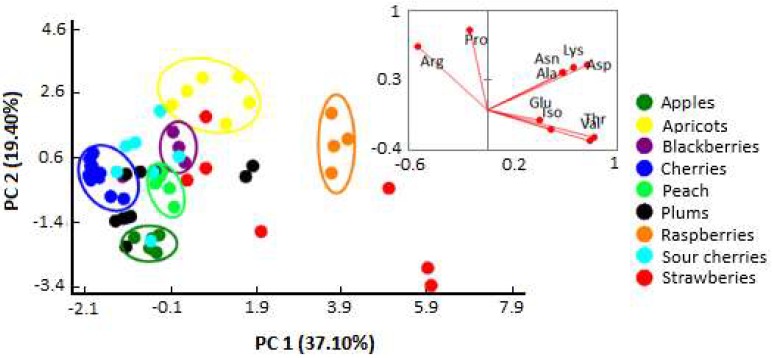
Scatter diagrams of a principal component analysis (PCA) based on the loading points of PC1 and PC2, including the fruit juice sample distribution map.

Fruit juices are natural products with a very complex matrix, so it is rather difficult to reach a conclusion by analyzing the data of all classes of compounds. Although previous tests showed that each sample had different free amino acid profile, chemometric approaches, such as principal component analysis (PCA) and linear discriminant analysis (LDA), can highlight additional differences and relationships. Thus, in order to realize a chemometric analysis, the content of 10 amino acids from 64 fruit juice samples (cherries, sour cherries, plums, apricots, peaches, apples, strawberries, raspberries, and blackberries) previously determined by ^1^H-NMR were imported into XLSTAT software for data mining. To evaluate the presence of latent variables correlating the single metabolites in the fruit juice samples for their botanical origin discrimination, PCA was applied to the intensity of 10 ^1^H resonances, representing the identified amino acids. This multivariate statistical technique achieves data reduction by a linear combination of the original variables, highlighting the variance within the original dataset, and retaining most of the relevant information of the variables in the new first components. The total four principal components extracted, whose eigenvalues exceeded 1, can explain 79.79% of the total variance, with the first and principal components (PC1 and PC2) accounting for 37.10% and 19.40%, respectively. Because PC1 and PC2 are significant and can explain as much as 56.50% of the total variance, this indicates that PC1 and PC2 are informative and could be applied with respect to clustering samples in two-dimensional space. To identity the relationship between the amino acid profile and the type of fruit, a biplot was performed (Figure 3). The first principal component (PC1) was strongly associated with the contents of Lys, Asp, Asn, Ala, Thr, and Val, the second principal component (PC2) was mainly associated with Pro and Arg, while the other amino acids (Glu and Iso) were associated with PC3 and PC4.

The application of PCA revealed some compositional similarities and helped to explore the overall variability in the population of samples, observing some defined and discriminated clusters of samples, such as apricots, blackberries, peach, cherries, apples, and raspberries. A relationship between the quantified amino acids and some fruit juices was also observed (Figure 3). Apricot and blackberry juices are mainly associated with Arg and Pro, strawberry and raspberry juices presented a strong positive correlation with Lys, Asp, Asn, Ala, Thr, and Val, while cherries juice presented a negative correlation with PC1 amino acids loadings, as well as is the case of apple juice with PC2 loadings.

**Figure 4:**
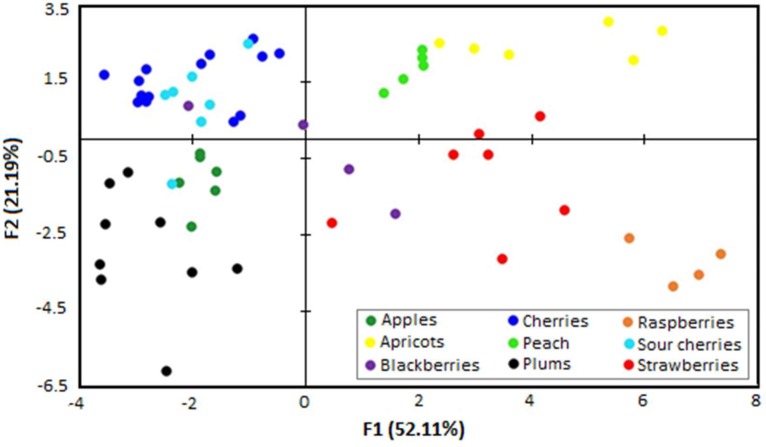
Scatter diagram of fruit juice samples analyzed according to different botanical origin, obtained through the two discriminant functions after linear discriminant analysis (LDA).

Based on the obtained statistical results, it can be ascertained that the amino acid profile of different types of fruit juices could provide valuable information regarding botanical origin. However, PCA is an unsupervised chemometric method that ignores class labels and does not represent a perfect or definitive technique that is able to determine the botanical origin of a fruit juice sample based only on its amino acids data. In this regard, to investigate the suitability of this approach for the classification of fruit juices samples according to their botanical origin, LDA was performed with the whole dataset (Figure 4).

LDA is a supervised multivariate statistical method used to identify the respective type of a sample according to various values, under a defined classification system [24]. Five discriminant functions were obtained by LDA, and all functions were demonstrated to be significant for correct classification. The first function accounted 52.11% of the total variance, with Wilks’ Lambda = 0.0006, X_2_ = 399.948, d_f_ = 80, and sig. = 0.000, while the second function accounted for 21.19% of the total variance, with Wilks’ Lambda = 0.006, X_2_ = 270.057, d_f_ = 63, and sig. = 0.000.

The first quadrant mainly includes cherries and sour cherries; apricot and peach fruit juices are mostly distributed in the second quadrant; apples and plums in the third; and strawberry, blackberry, and raspberry fruit juices in the fourth quadrant. A possible explanation for this separation may be related to a mixing of multiple sources of variation such as the different harvest season (e.g., rainfall, temperature means) as well as the genus of the plant from which the fruits originate. It can be observed that most of the “superfruit” juices, produced from fruits that have exceptional nutritional quality, such as various types of berries (e.g., strawberries, blackberries, and raspberries), have a relatively similar distribution in the scatter plot. Function 1, which provides the main separation between the botanical origins of fruit juices, was primarily correlated with Lys, Thr, and Val while the second function was correlated with Pro, Asn, Asp, and Iso. The variables selected by the discriminant analysis as the most powerful for differentiation were, in this order, Lys, Asp, Ala, and Iso.

The results of the LDA are superior, and according to the confusion matrix for the estimation sample, a 93.75% was reached (one sample of blackberry juice was classified as sour cherry juice, one sour cherry juice was classified as apple juice, and two strawberry juices were appointed in other categories—Table 2).

## 3. Materials and Methods

### 3.1. Fruit Juice Samples

The study was conducted on 64 fruit juices samples from Rosaceae family, including nine different varieties from four genera (Table 3), namely: Cherries (*Prunus avium*), sour cherries (*Prunus cerasus*), plums (*Prunus domestica*), apricots (*Prunus armeniaca*), peaches (*Prunus persica*), apples (*Malus domestica*), strawberries (*Fragaria sp.*), raspberries (*Rubus idaeus*), and blackberries (*Rubus fruticosus*), grown in 3 different geographical regions from Romania (Oltenia–Valcea county, Dobrogea–Constanta county, and Moldova–Vrancea county, situated in the South-West Oltenia region, South-East, and East of Romania, respectively). The fruits were harvested at commercial maturity and the collected fruit samples were cooled and transported to the laboratory, assuring the maintenance of the cold chain. Subsequently, fruits were washed with water, kept frozen, and stored at −20 °C in a freezer.

### 3.2. Sample Preparation for NMR Analysis

Approximately 50 g of macerated fruits were centrifuged (12,000× *g*, 20 min) and filtered through a 45 µm disposable filters, yielding about 10 mL of juice. All juice samples were pH-adjusted to 2.65 ± 0.02 using 5 N HCl and 5 N NaOH solutions. A volume of 700 μL from each sample was mixed with 70 μL of D_2_O (as a field frequency lock signal) containing 0.2% *w/w* of 3-(trimethylsilyl)propionic-2,2,3,3-d4 acid, sodium salt (TMSP as an internal standard), and sodium azide (0.013% *w/w*) to suppress microorganism activity, and was transferred to a 5-mm NMR tube. Before the analysis, no additional treatment was necessary. All the fruit juice samples were analyzed in two replications. All chemical reagents were purchased from Sigma-Aldrich (St. Louis, MO, USA).

### 3.3. NMR Analysis

All the measurements were performed on a Bruker Avance III 400 NMR spectrometer (Bruker France SAS, Wissembourg, France) operating at 9.4 T, equipped with a 5 mm BBO probe, observing ^1^H at 400 MHz and automatic tuning-matching (ATM). A time delay of 5 min was set between sample injection and pre-acquisition calibrations to ensure complete temperature equilibration. Experiments were run at 300 K in automation mode after loading individual samples on a Bruker Automatic Sample Changer, interfaced with the software Icon NMR (Bruker). The suppression of H_2_O signals was carried out by using noesygppr-1d (Bruker standard pulses sequence), applying continuous waves during the relaxation delay (2.0 s) with a mixing time of 10.5 µs. The ^1^H-NMR spectrum was measured over a spectral width of 6402 Hz, with 32,768 data points, acquisition time of 2.559 s, and 8 scans. All spectra were processed (line broadening, Fourier transform, phase correction, and baseline adjustment), by using the standard routines of the Bruker software TopSpin version 3.2. The resulting spectra were aligned using the TMSP signal as reference and the results were represented by the intensity of the peaks produced by the amino acid groups in the spectral regions 0–3.4 ppm and 5.5–10 ppm, as a percentage of the total signals produced by the organic groups in the sample. This normalization was performed in order to compare the samples, which show different concentrations of compounds related to the water content. The obtained signals were correlated for all samples by aligning the values for the same identified peak. Peaks assignment was performed by means of literature data [3,18,25,26,27,28,29,30,31,32].

### 3.4. Software and Data Pre-Processing

Appropriate multivariate statistical tools were applied to extract meaningful information from such a large data set, and usually big efforts are made to give a biological interpretation of the results. Because of the existence of different factors (e.g., botanical and geographical origin,) that could affect the fruit composition, the principal component analysis (PCA) and linear discriminant analysis (LDA) was applied to the ^1^H-NMR data in order to evaluate the variables capable of characterizing the fruit juices origin. In this context, we made a data matrix in which the rows represent the different fruit juice samples analyzed and the columns correspond to the ^1^H-NMR data [33]. These data were processed by LDA which is a powerful technique for pattern recognition that attempts to explain the variance of a large set of inter-correlated variables. LDA transforms the original data in a set of “scores” for each sample, measured by discriminant component axes. Scattering diagrams of the scores of the first principal components used provides an excellent view and sometimes gathering together to show similar evidence, separating different types of samples or samples with different values of this selection. Cross-validation was applied to determine the optimal number of variables required to obtain robust models. All the mathematical and statistical processing of data were performed using commercial software packages as Microsoft Excel 2013 (Microsoft, Redmond, WA, USA) and XLSTAT Addinsoft 2014.5.03 version (Addinsoft Inc., Long Island, NY, USA).

## 4. Conclusions

This study used a ^1^H-NMR-based metabolomics approach coupled with multivariate statistical analysis to demonstrate the powerfulness of this technique in differencing the botanical origin of fruit juices based on its amino acid profile. Relative amino acid profiles differ among different fruit juice samples depending on the fruit variety, and the observed deviations have the potential to support their quality and authenticity/botanic origin assessment. Non-targeted NMR amino acid metabolomic fingerprinting proved to be the most suitable methodology to obtain “high-throughput” spectroscopic data on a wide range of compounds with a high analytical precision. In this study, several amino acids were identified in nine different fruit juices from four genera, and their relative amounts have permitted a comprehensive view of the investigated varieties, allowing the amino acid metabolite profiles to be mapped as completely as possible. Those compounds playing key roles in the observed physiological variation or specific bioassays were then identified using statistical analyses (e.g., principal component and/or linear discriminant analysis). Our results showed that this information could be applied for a rapid classification of fruits based on their varietal origin and to further develop and build a model capable of detecting adulterations of fruit juices.

## Figures and Tables

**Table 1:** Correlation analysis for amino acids profile and botanical origin.

Variables	Pro	Arg	Lys	Asn	Asp	Glu	Iso	Ala	Thr	Val
Genus										
*Prunus*	0.455	0.596	−0.220	−0.239	−0.436	−0.083	−0.116	−0.363	−0.470	−0.422
*Fragaria*	−0.147	−0.360	0.347	0.199	0.324	0.181	0.275	−0.034	0.616	0.631
*Malus*	−0.576	−0.503	−0.272	−0.019	−0.099	−0.147	−0.177	−0.180	−0.109	−0.110
*Rubus*	0.001	−0.063	0.224	0.168	0.400	0.076	0.061	0.706	0.182	0.100

**Table 2:** Confusion matrix for the fruit variety estimation.

From\To	Apple	Apricot	Blackberry	Cherry	Peach	Plum	Raspberry	Sour Cherry	Strawberry	Total	% Correct
Apple	6	0	0	0	0	0	0	0	0	6	100%
Apricot	0	6	0	0	0	0	0	0	0	6	100%
Blackberry	0	0	3	0	0	0	0	1	0	4	75%
Cherry	0	0	0	15	0	0	0	0	0	15	100%
Peach	0	0	0	0	5	0	0	0	0	5	100%
Plum	0	0	0	0	0	9	0	0	0	9	100%
Raspberry	0	0	0	0	0	0	4	0	0	4	100%
Sour cherry	1	0	0	0	0	0	0	7	0	8	87.5%
Strawberry	0	1	0	0	0	1	0	0	5	7	71.43%
Total	7	7	3	15	5	10	4	8	5	64	93.75%

**Table 3:** Description of fruit samples collection.

Fruit Variety	Fruit Genus	Fruit Species	Harvest Period	Region of Provenance		
				Valcea County	Constanta County	Vrancea County
Apricots	*Prunus*	*P. armeniaca*	August	2	2	2
Cherries		*P. avium*	June	5	5	5
Peach		*P. persica*	August	2	2	1
Plums		*P. domestica*	August	3	3	3
Sour cherries		*P. cerasus*	June	3	2	3
Raspberries	*Rubus*	*R. idaeus*	August	1	2	1
Blackberries		*R. fruticosus*	August	2	1	1
Strawberries	*Fragaria*	*Fragaria sp.*	June	3	2	2
Apples	*Malus*	*M. domestica*	August	2	2	2
